# Subpopulation dynamics of T and B lymphocytes in Sjögren’s syndrome: implications for disease activity and treatment

**DOI:** 10.3389/fimmu.2024.1468469

**Published:** 2024-09-03

**Authors:** Qingliang Meng, Junfu Ma, Jiakang Cui, Yangyi Gu, Yu Shan

**Affiliations:** ^1^ Department of Rheumatism, Henan Province Hospital of Traditional Chinese Medicine, Zhengzhou, Henan, China; ^2^ Guanghua Clinical Medical College, Shanghai University of Traditional Chinese Medicine, Shanghai, China

**Keywords:** Sjögren’s syndrome, pathogenesis, T lymphocytes, B lymphocytes, targeted therapy

## Abstract

Sjögren’s syndrome (SS) is an autoimmune disorder primarily affecting the body’s exocrine glands, particularly the salivary and lacrimal glands, which lead to severe symptoms of dry eyes and mouth. The pathogenesis of SS involves the production of autoantibodies by activated immune cells, and secretion of multiple cytokines, which collectively lead to tissue damage and functional impairment. In SS, the Immune interaction among T and B cells is particularly significant. Lymphocytic infiltration in the salivary glands is predominantly composed of CD4+ T cells, whose activation cause the death of glandular epithelial cells and subsequent tissue destruction. The excessive activity of T cells contributes significantly to the disease mechanism, with helper T cells (CD4+) differentiating into various subgroups including Th1/Th2, Th17, as well as Treg, each contributing to the pathological process through distinct cytokine secretion. In patients with SS, B cells are excessively activated, leading to substantial production of autoantibodies. These antibodies can attack self-tissues, especially the lacrimal and salivary glands, causing inflammation and tissue damage. Changes in B cell subpopulations in SS patients, such as increases in plasmablasts and plasma cells, correlate positively with serum autoantibody levels and disease progression. Therapies targeting T cells and B cells are extensively researched with the aim of alleviating symptoms and improving the quality of life for patients. Understanding how these cells promote disease development through various mechanisms, and further identifying novel T and B cell subgroups with functional characterization, will facilitate the development of more effective strategies to treat SS.

## 1 Introduction

Sjögren’s Syndrome (SS) is a systemic autoimmune disorder characterized by impaired salivary and lacrimal gland function, causing pronounced dryness of the skin and eyes (1). Aside from the classic symptoms of dryness, fatigue, and joint pain, SS can impact various organs, manifesting symptoms in the lungs and neuropsychiatric domains (2). Furthermore, research suggests a heightened susceptibility to cardiovascular diseases and lymphoma in individuals with SS (3). SS is a prevalent rheumatic disorder, with an estimated prevalence of 0.5%, an annual incidence ranging from 3 to 11 cases per 100,000, and an estimated mortality rate of 4 per 1,000 per year (4). Women exhibit a higher incidence rate, with a gender ratio of nearly 10:1 (5). Gender differences in disease presentation are evident, with men often experiencing more severe eye involvement and less pronounced systemic and immune symptoms (6). There is no age limit on when SS manifests, although it primarily impacts individuals between the ages of 30 and 50, thus making its occurrence in children uncommon (7). This autoimmune disease is associated with significant mortality rates, largely attributed to complications such as B-cell lymphoma, interstitial lung disease, renal failure, and severe cryoglobulinemic vasculitis. Notably, non-Hodgkin lymphoma stands out as one of the most severe complications (8).

The exact etiology and pathogenesis of SS are currently unknown, with the condition thought to arise from intricate interactions among the activated immune system, epithelial cells, and target cells implicated in the autoimmune response, influenced by various factors crucial to disease progression (9). Genetic factors, particularly those identified through genome-wide association studies (GWAS), are of primary importance, with additional research indicating the involvement of other risk alleles (10). Wang and colleagues discovered 39 immune-related gene variants using whole-exome sequencing in families affected by primary SS, with a predominant association with the activation of T cell as well as their receptors (11). The dysregulation of the immune system and progression of the disease may be attributed to epigenetic modifications such as DNA methylation and histone modifications. It has been identified that IFN-regulated genes were hypomethylated in salivary gland (SG) cells and immune system of individuals with SS (12, 13). Besides genetic influences, immune dysregulation, environmental factors, infections, and medications have the potential to disrupt both innate and adaptive immune responses, leading to the induction of interferon signaling and subsequent stimulation of B cell proliferation (14). Furthermore, neuroendocrine mechanisms involving hormones and neuropeptides may impact exocrine gland function, providing a potential explanation for the presence of severe dryness symptoms in some patients with SS despite the absence of prominent inflammatory histopathological features (15).

The main pathological characteristics of SS include SG lymphocytic invasion and the presence of autoantibodies in the bloodstream. Infiltrating lymphocytes, predominantly T and B cells, tend to aggregate around striated duct lesions, although other immune cells such as dendritic cells, may observed as well (16, 17). Research indicates that the lymphocytes infiltrating glandular tissues are primarily composed of CD4+ T cells, with some CD8+ T cells present. Activation of these T cells leads to epithelial cell death and tissue destruction (18). Furthermore, the infiltration of T cells into exocrine glands and other tissues initiates inflammatory responses, resulting in the symptomatic presentation of SS (19). Regarding autoantibodies, most individuals suffering from SS have antibodies against SSA/Ro and SSB/La (20), which has a positive association with the number of corresponding plasma cells in the SGs. Based on this, it seems that the SGs serve as a significant reservoir for autoantibody-producing cells (21). These autoantibodies are produced by activated B lymphocyte as well as plasma cells. The heightened activity of B lymphocyte leads to the production of autoantibodies and various cytokines, with emerging evidence indicating the functional heterogeneity of B lymphocyte subsets in both immune reactions and autoimmune pathogenesis (22). This review emphasizes the significance of immune cell activation on SS and outlines the key pathways through which T and B lymphocytes, as well as their subsets, contribute to the development of SS. Comprehending these intricate immune pathways will aid in the investigation of the effective targeting of particular immune cells for therapeutic purposes.

## 2 T lymphocytes

**Figure 1:**
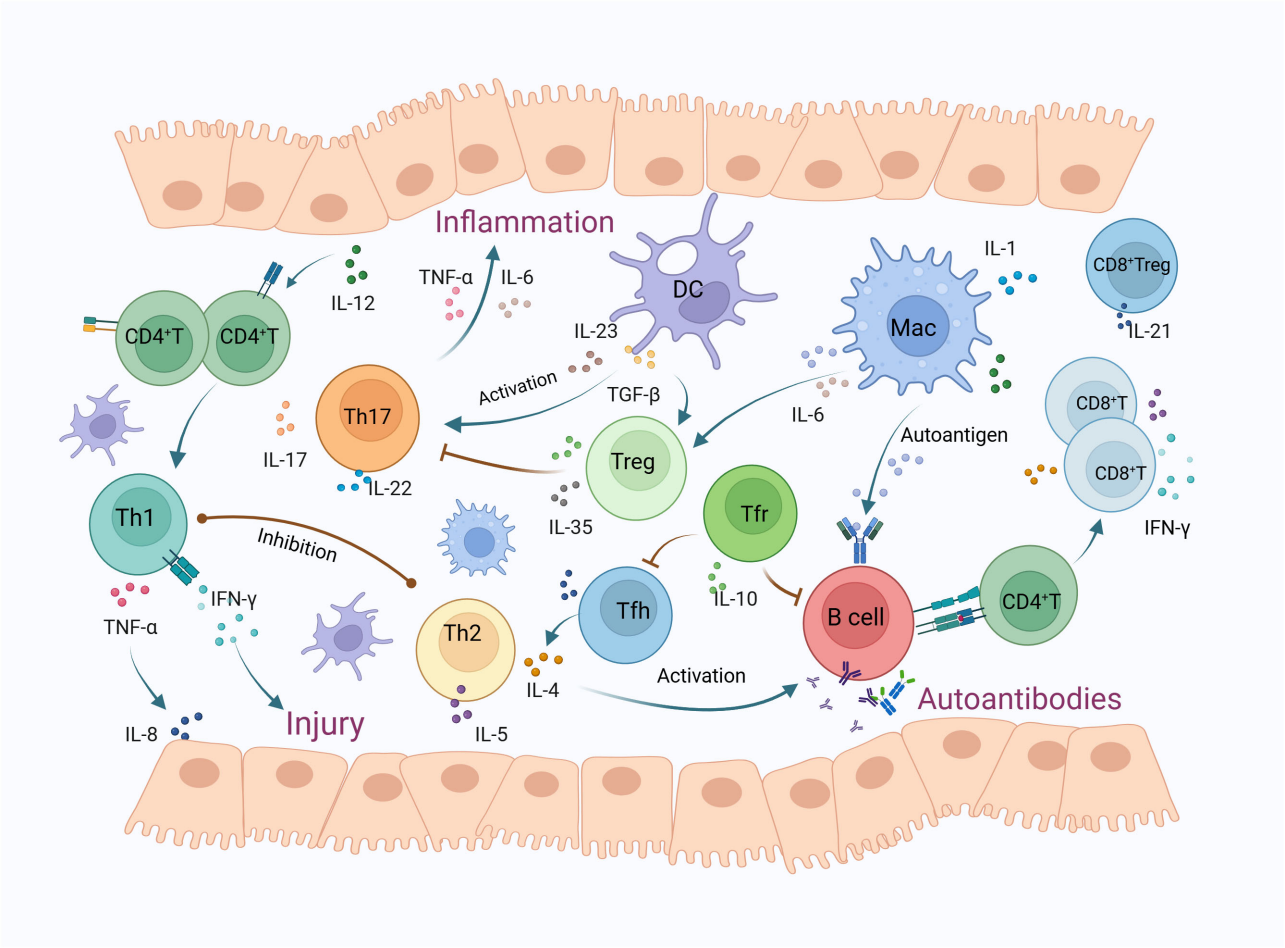
The mechanisms of T lymphocytes and their subsets in Sjögren’s syndrome. T lymphocyte infiltration in the salivary glands of SS patients has increased, predominantly consisting of CD4+ T and CD8+ T cells. IL-12 activates CD4+ T cells to differentiate into Th1 cells, releasing cytokines such as IFN-γ and TNF-α, which cause glandular damage and inflammation. TNF-α also triggers the release of IL-8, thereby enhancing leukocyte infiltration. Th2 cells secrete IL-4 and IL-5 to activate B cells, participating in the formation of autoantibodies, while also inhibiting Th1 cells. DCs release TGF-β and IL-23, promoting Th17 cell polarization. Activated Th17 cells induce inflammatory exocrine glands to secrete IL-6 and TNF, leading to inflammation. TGF-β can also activate Tregs, which secrete inhibitory cytokines such as IL-10 and IL-35, thus inhibiting Th17 cell responses, whereas IL-6 can mediate the conversion of Tregs to Th17 cells. Macrophages play a role in antigen presentation, activate B-cell-mediated humoral immunity, and also secrete cytokines such as IL-6 to participate in inflammatory responses. Tfh cells primarily secrete IL-21 and IL-4, promoting B cell proliferation and differentiation within germinal centers, ultimately producing high-affinity antibodies. Tfr cells can effectively inhibit Tfh and B cells, thus regulating germinal center reactions and the production of autoantibodies. CD8+ T cells secrete inflammatory cytokines such as IFN-γ and TNF-α, and cause salivary gland cell apoptosis through the FAS and CTL pathways. CD8+ Tregs primarily release IL-21, inhibiting immune responses by suppressing lymphocyte function. (DC, dendritic cell; MAC, macrophages; Treg, regulatory T cells; Tfh, follicular helper T cells; Tfr, follicular regulatory T cells).

SS is commonly recognized as a disorder primarily activated with T cells, as evidenced by the predominance of T cell infiltrates in early-stage lesions (23). Research has revealed an increase in T cell infiltration within the SGs of individuals with SS, while T cell levels in peripheral blood are decreased, likely due to migration, and are associated with disease progress (24). These infiltrating lymphocytes are primarily comprised of helper T cells (Th), which can differentiate into various subsets including Th1, Th2, Th17, regulatory T cells (Treg), and Tfr, Tfh, as well as CD8+ T cells (25). The pathogenesis of primary SS is significantly influenced by the heightened activity of T cells, which persistently trigger local inflammatory immune reactions through the innate immunity (**Figure 1**).

### 2.1 Th1/Th2 cells

The Th1 cells respond to immune stimuli by releasing cytokines such as interferon-gamma (IFN-γ), tumor necrosis factor-alpha (TNF-α), interleukin-2 (IL-2), and C-X-C motif chemokine receptor 3 (CXCR3) (26). In patients with SS, the activation of Th1 cells results in damage to glandular tissue and inflammation. These cytokines serve to recruit additional inflammatory cells to the affected areas, thereby intensifying the inflammatory response (27). Researchers have previously demonstrated high levels of IFN-γ, TNF-α, and IL-4 in the saliva of SS patients, suggesting a correlation between heightened Th1-related inflammatory cytokines and the development of SS (28). IFN-γ, a prominent cytokine produced by Th1 cells, is essential in modifying the activation of specific innate immune cells. Excessive activation of IFN-γ may exacerbate the pathology of SS (29). This cytokine has been shown to impair tight junctions in the glandular tissues of individuals with SS, leading to dysfunction of the glands result from the present of local inflammatory cytokines (30). TNF-α, another Th1 cytokine, is also linked to the progression of SS, as elevated levels have been detected in the serum with the condition and significantly increased in their saliva and SGs (31). TNF-α facilitates leukocyte adhesion through the modulation of epithelial junction reorganization and the upregulation of adhesion molecule expression (32). Additionally, TNF-α triggers the release of interleukin-8 (IL-8), which enhances leukocyte infiltration and contributes significantly to the pathogenesis of SS (33). Furthermore, studies have identified elevated levels of interleukin-12 (IL-12) in the SGs of patients with SS (34). Suboptimal concentrations of IL-12 have been shown to activate CD4+ T cells to differentiate into Th1 cells via the activation of the STAT4 phosphorylation pathway (35). It has been demonstrated that IL-12 stimulate IFN-γ production and promote Th1 cell differentiation, with decreased levels of IL-12 correlating with reduced symptoms in mouse models (36). IL-27, a cytokine related to IL-12, has the ability to drive the differentiation of various immune cell types, including T, B, NK, and dendritic cells. Elevated levels of IL-27 in the peripheral blood of individuals with SS might contribute to the differentiation of Th1 cells, leading to alterations in the Th1/Treg cell balance as well as the activation of T cells, ultimately contributing to the development of SS symptoms (23).

Th2 cells are known for their secretion of cytokines like interleukin-4 (IL-4), interleukin-5 (IL-5), interleukin-13 (IL-13), which are essential component of humoral immunity (37). In contrast, Th1 cells primarily produce IFN-γ to support cellular immune responses and suppress Th2 cell activity. Excessive inflammation can result in uncontrolled tissue damage. In addition to producing IL-4 and IL-5, Th2 cells stimulate IgE production and eosinophil activation, while inhibiting Th1 responses (38). Hence, maintaining a delicate equilibrium between Th1 and Th2 cells is essential for immune regulation. Th2’s activation is particularly significant in SS, as relevant cytokines induce B cell hyperactivation and the production of antibodies, including anti-SSA/Ro and anti-SSB/La (39). The hyperactivation of B cells and the production of autoantibodies contribute to SS pathogenesis. Research indicates an increase in Th2 cells in the peripheral blood of SS patients, which contributes to the progression of the disease (40). Furthermore, elevated levels of the Th2-derived cytokine IL-10 in saliva are associated with disease progression in a positive manner (41). Research has identified the presence of IL-2 and IFN-γ in all patients with SS, while IL-4 and IL-5 are exclusively present in individuals exhibiting elevated levels of B cell infiltration in the SGs (42). Clinical evidence suggests a marked elevation of Th2 cytokines in the saliva of SS patients, with Th2 cytokine levels demonstrating a strong correlation with heightened lymphocyte accumulation in the labial SGs (43). Immunohistochemical analysis demonstrates the localization of Th2 chemokines, such as C-C motif chemokine ligand 22(CCL22) and C-C motif chemokine ligand 17(CCL17), in the vicinity of ductal epithelial cells and germinal centers (43). These findings indicate the crucial function of Th2 cytokines at the beginning and perpetuation of SS, particularly in the context of localized B cell activation.

### 2.2 Th17 cells

Th17 cells express interleukin-17 (IL-17) and interleukin-22 (IL-22), as well as chemokine receptors CCR6, CCR4. Th17 contribute significantly to the pathogenesis of various autoimmune diseases (44, 45). Additionally, IL-17 and IL-22 produced by Th17 promote the formation of tight junction proteins, thereby contributing to the maintenance of epithelial barrier integrity. Epithelial cell survival and proliferation are also significantly influenced by IL-22 (46, 47). Located in the lymph nodes of the salivary glands and the lacrimal glands, dendritic cells may initiate and polarize Th17 cells. Subsequently, in the advanced stages of the disease, these dendritic cells release transforming growth factor-β(TGF-β) and IL-23 to facilitate Th17 cell polarization (45). The activated Th17 cells contribute to inflammation by inducing inflamed exocrine glands to secrete IL-6 and TNF (48). There is a notable increase in Th17 cells in the SS patients’ peripheral blood, and the levels of IL-17 in their saliva and tears surpass those of healthy individuals (49). According to Reksten et al (50), the serum IL-17 levels were higher in small SG biopsies from germinal centre (GC)-positive patients (patients with ectopic GC formation in the salivary glands) than in GC-negative patients, and there was a positive correlation among serum IL-17 levels and anti-Ro/SSA as well as anti-La/SSB. Additionally, an increase in circulating Th17 was only found in SS individuals with moderate to high disease activity, suggesting a relationship between the number of circulating Th17 cells, serum IL-17 levels, and disease severity (51). Conversely, elevated IL-22 expression promotes the aggregation of B cells and the formation of lymphoid aggregates, resulting in the upregulation of multiple cytokines including CXCL12 and CXCL13, ultimately leading to the production of autoantibodies (52). Increased IL-22 is linked to the anti-SSB autoantibodies, combined anti-SSA/SSB antibodies, rheumatoid factor, and reduced saliva flow rate in patients with SS (53).

### 2.3 Regulatory T cells

Regulatory T cells (Tregs) express the surface markers CD4, CD25, and the transcription factor Forkhead Box P3 (Foxp3) that distinguish them from suppressive T cells (54). Tregs have a crucial function in preventing the development of autoimmune and allergic ailment through the secretion of inhibitory cytokines like IL-10, IL-35, TGF-β, as well as the induction of cell lysis (granzyme B/A), which modulates the activation and function of antigen-presenting cells (55). Both Tregs and Th17 cells could be activated by TGF-β, and the delicate balance between these cell subsets can be easily disrupted, resulting in the predominance of pathogenic cells and the initiation of autoimmune responses (56). An imbalance in the ratio of Th17 cells to Tregs has been documented in several autoimmune conditions, such as inflammatory bowel disease, rheumatoid arthritis, and multiple sclerosis (57–59). In patients with SS, there has been a reduction in the proportion of CD4+CD25+ Treg cells in the bloodstream, alongside elevated levels of TGF-β in the SGs, potentially attributed to the IL-6-mediated transformation of Tregs into Th17 (60). Moreover, a particular subgroup of circulating CD4+ T cells with reduced CD25 expression has been identified in patients with SS. These cells, emergence in the SGs, demonstrate a regulatory T cell (Treg) phenotype by expressing Foxp3, TGF-β, and IL-10, despite their little expression of CD25. They proliferate exclusively in individuals with quiescent disease and display potent suppressive capabilities against self-reactive cells (61). In contrast to conventional Tregs, another regulatory subset known as type 1 regulatory T (Tr1) cells, induced by IL-27, exhibit heightened IL-10 production and the ability to dampen T cell responses. The prevalence of Tr1 is notably diminished in individuals with SS and in mouse models of the disease (62).

### 2.4 Tfh/Tfr cells

Tfh cells, an identifiable subset of CD4+ helper T cells, are predominantly situated in the lymphoid follicles of secondary lymphoid organs such as tonsils, spleen, and lymph nodes, which are characterized by the expression of phenotypic markers including CXCR5, CD40L, ICOS, Bcl-6, and PD-1 (63). Tfh cells primarily secrete IL-21 and IL-4 to proliferate B cells and differentiation within germinal centers, ultimately resulting in developing high-affinity antibodies. This process is essential for the maintenance of germinal center activity and humoral immune balance (64). Research has indicated an increase in Tfh cells in the SS patients’ peripheral blood and SGs, with these levels showing a positive correlation with the Sjögren’s Syndrome Disease Activity Index (ESSDAI) (65). Circulating Tfh cells (cTfh) exhibit distinct characteristics from germinal center Tfh cells, notably the absence of BCL-6 expression. Specifically, cTfh1 cells are known to release IL-21, IL-10 as well as IFN-γ; cTfh2 cells emergence IL-4, IL-13, and IL-21; and cTfh17 cells secrete IL-17, IL-21, IL-22 (63). Of these, cTfh17 cells exhibit higher expression levels in SS individuals and demonstrate a positive link to disease activity, total IgG levels, and the amount of anti-SSA/anti-SSB (66). Inducible T-cell costimulator (ICOS) serves as an active biomarker for cTfh. Research indicates that ICOS expression in cTfh cells of SS patients is three times greater than in healthy individuals, and ICOS is upregulated in conjunction with CXCR5 and PD-1, resulting in heightened activation of Tfh cells in SS patients (67).

Follicular regulatory T cells (Tfr) are a distinct subset of Tregs that express Foxp3, CXCR5, and Bcl-6. These cells migrate to B cell follicles and GCs in which they play a vital function in inhibiting the overactivation of Tfh and B cells, thus preventing autoimmune responses (68). Similar to Tfh cells, Tfr cells exhibit high expression of CXCR5 while also retaining the Foxp3 on the surface of Tregs. This allows Tfr cells to effectively inhibit Tfh and B cells, thereby regulating GC reactions and autoantibodies production (69, 70). Tfr exhibit a notable increase in abundance within the blood and SGs of SS, with a significantly higher Tfr/Tfh ratio observed in their blood compared to that of healthy individuals (71). Moreover, circulating Tfr cells (cTfr) have garnered significant attention in SS research, with key markers including Foxp3, CXCR5, and CD4. Studies have demonstrated a correlation between the cTfr/cTfh ratio and the development of ectopic lymphoid structures and lymphocyte infiltration in the SGs of SS (65). Additional studies demonstrate that Tfr cells present in the bloodstream serve as markers for continuous humoral activity. Elevated levels of cTfr cells could contribute to active lymphocyte proliferation and differentiation, as opposed to glandular inflammation (72).

### 2.5 CD8+ T cells

CD8+ T cells, or cytotoxic T cells, serve a critical function in the human immune system by recognizing antigens presented by MHC class I molecules through their CD8 receptors and releasing cytotoxins to eliminate target cells (73). Recent research indicates a notable enrichment of CD8+ T cells in the SGs of those suffering from SS, accompanied by a decrease in their numbers in peripheral blood, potentially attributed to the migration and accumulation of circulating CD8+ T cells in the glands (74). CD8+ T cells are observed to engage in direct cytotoxic activity against affected glandular cells, resulting in the manifestation of classic symptoms of SS such as xerostomia and xerophthalmia (23). Notable subtypes of CD8+ T cells include type 1 CD8+ T cells (Tc1), Tc2, Tc17 cells, and regulatory CD8+ T cells (CD8+ Tregs). Tc1 are characterized by the release of IFN-γ and TNF-α, indicating potent cytotoxic capabilities. Tc2 cells predominantly generate IL-4, IL-5, and IL-13. Tc17 secrete IL-17 and are implicated in inflammatory responses and autoimmune conditions. CD8+ Tregs primarily release IL-21, which serves to dampen immune reactions by inhibiting lymphocyte functions (75). In non-obese diabetic (NOD) mice deficient in CD8+ Tregs, an increase in Th17 cells results in corneal damage and exacerbated SS pathology. Therefore, the depletion of CD8+ Tregs may play a significant role in SS’s development (76). Studies have revealed that CD8+ T cells within the SGs of SS patients exhibit heightened activity and abnormal proliferation, leading to the accumulation of memory CD8+ T cells and elevated production of cytokines that cause inflammation, like IFN-γ and TNF-α. Within the spleen and SGs of NOD mice, CD8+ T cells clump together and use the FAS and CTL pathways to cause SG cells to undergo apoptosis (77).

### 2.6 Innate T cells

Innate T cells, including gamma delta (γδ) T cells, mucosal-associated invariant T (MAIT) cells, and natural killer T (NKT) cells, recognize antigens in a manner unrestricted by classical major histocompatibility complex (MHC) class I or II and respond rapidly upon activation (78). A subpopulation of T lymphocytes known as γδT cells is distinguished by the expression of altered γ and δT-cell receptors on their surfacea (79). As innate immune cells, γδT cells rapidly recognize exogenous pathogens and endogenously stressed-induced ligands in an MHC-unrestricted manner, triggering adaptive immunity as the first line of immunological defense (80). Compared to healthy individuals, the frequency of γδT cells in the blood of patients with SS is increased (81), and they play a supportive role in inducing B cells to secrete immunoglobulins (82). Studies report that γδT cells, especially Vγ4 T cells, are involved in the pathogenesis of SS and SS-related pulmonary inflammation. In NOD mice, an increase in γδT cells and their subgroups (Vγ4 IL-17A T cells) in the lungs and spleen reduces saliva flow rate and exacerbates pulmonary pathology (83).

MAIT cells are unconventional innate-like T cells, activated through binding with the MR1 molecule, a class I MHC-like entity (84). MAIT cells are abundant in peripheral blood, liver, gastrointestinal tract, and mesenteric lymph nodes, playing a crucial role in mucosal immunity against infections by rapidly producing cytokines such as IFN-γ, IL-21, TNF-α, IL-17, perforin, and granzyme B (85, 86). In patients with SS, MAIT cells are significantly reduced in peripheral blood but increased in salivary gland tissues (87), with elevated IL-17 levels produced by salivary gland MAIT cells (88). Immunohistochemical results show that MAIT cells are present in the labial salivary gland biopsy tissues of most SS patients but absent in those with mild sialadenitis unrelated to SS. Homing of MAIT cells to the glands may account for their reduced frequency in peripheral blood (87). Furthermore, the increased proportion of MAIT cells expressing CCR9 and CXCR5 in SS patients, as well as the overexpression of the ligands CCL25 and CXCL13 to promote their migration into inflamed tissues, suggests that these innately characterized cells may contribute to the immunopathology of SS (89).

NKT cells are a subgroup of innate T lymphocytes with characteristics of both T cells and natural killer (NK) cells, including effective TCR-mediated and NK-like cytotoxicity, producing abundant IFN-γ and TNF-α (90). Serving as a bridge between innate and adaptive immunity, NKT cells are associated with autoimmune diseases (91). Matthew and colleagues observed that the frequency of peripheral NKT cells in patients with systemic lupus erythematosus is low and negatively correlated with IgG levels, suggesting that NKT cells may regulate immunoglobulin production (92, 93). Compared to healthy controls, the number of peripheral blood NKT cells is significantly reduced in patients with primary Sjögren’s syndrome, but there is a considerable accumulation of NKT cells in the minor salivary glands of SS patients, correlated with the severity of sialadenitis, and high levels of IFN-γ secreted by NKT cells may exacerbate inflammation in the labial glands of SS patients (94, 95). In SS patients, NKT cells are significantly reduced in the periphery and infiltrate into the labial glands, possibly mediated by the CX3CL1-CX3CR1 chemotactic axis, with elevated CX3CL1 levels in the glands activating NKT cells and leading to high secretion of IFN-γ and TNF-α, thus contributing to the pathogenesis of SS (96). However, some research groups report an increase in NKT cells in SS patients. Discrepancies between these studies may be due to variations in the disease course and background treatment of the patients studied (97, 98). In a study on the treatment of SS patients, it was found that after 3 or 6 months of drug therapy, both the number and proportion of NKT cells in patients significantly increased (99).

## 3 B lymphocytes

**Figure 2:**
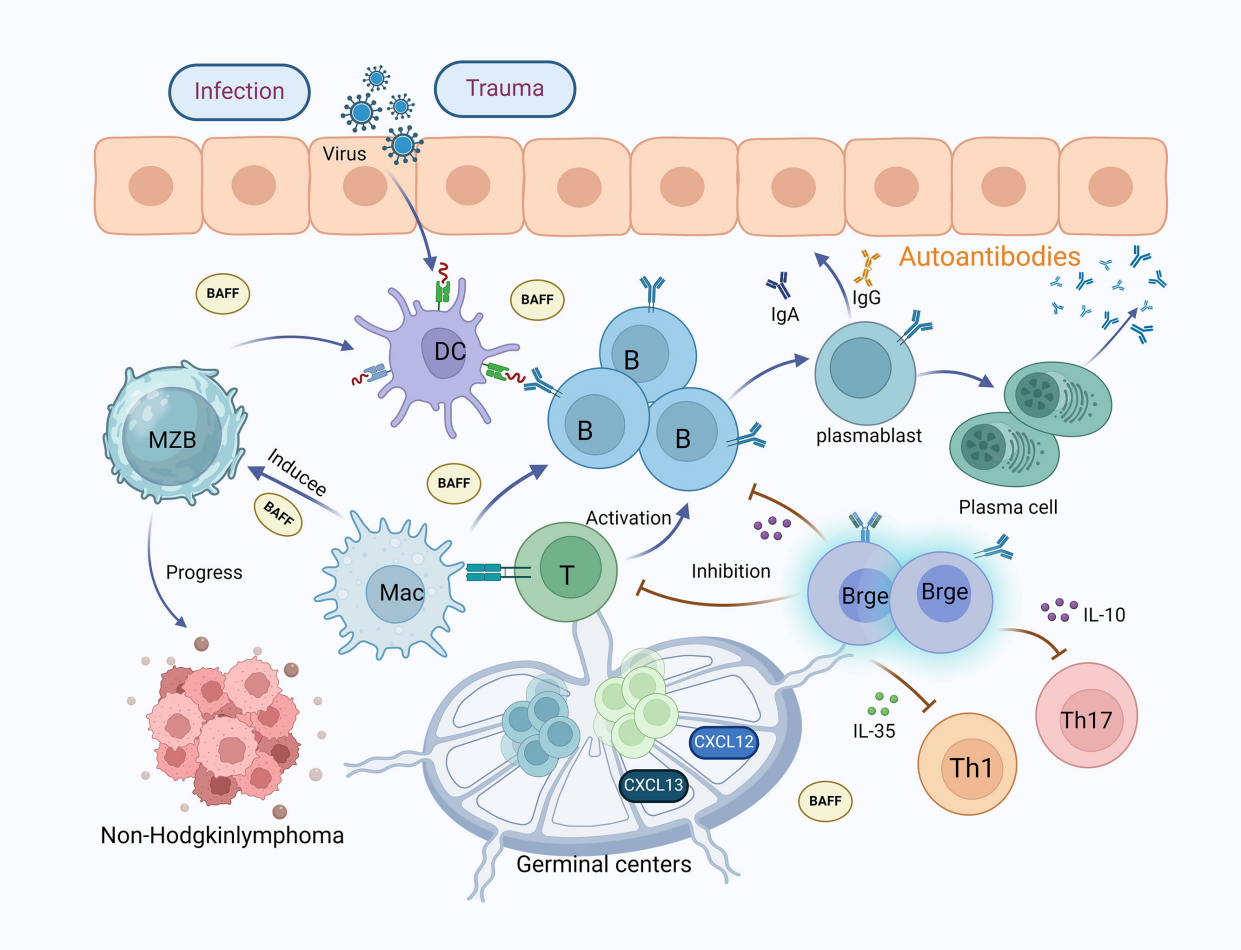
The mechanisms of B lymphocytes and their subsets in Sjögren’s syndrome. B lymphocytes are crucial to the pathophysiology of SS; increased activity of B lymphocytes leads to the production of autoantibodies, causing inflammation and tissue damage in the lacrimal and salivary glands. Viral, infectious, traumatic, and environmental factors stimulate dendritic cells, which in turn activate the proliferation and differentiation of B cells into plasmablasts and plasma cells, producing autoantibodies. MZB cells accumulate in the salivary glands of SS patients and assist in gland destruction by producing autoantibodies, being a primary source of non-Hodgkin’s lymphoma. Macrophages promote the activation of MZB cells and T cells, secreting large amounts of BAFF, which induces the proliferation and differentiation of B cells, thereby producing numerous antibodies. In SS patients, the lips and parotid glands accumulate ectopic lymphoid structures of secondary lymphoid organs (germinal centers), expressing chemokines CXCL12 and CXCL13, which aid in the proliferation of T and B cells. Furthermore, Bregs inhibit T and B cells and can directly suppress Th1 and Th17 cells by releasing IL-35 and IL-10. (MZB, marginal zone B cells; MAC, macrophages; DC, dendritic cell; Breg, regulatory B cells; BAFF, B-cell activating factor).

B lymphocytes are essential to the pathophysiology of SS, as their heightened activity can result in the generation of autoantibodies including rheumatoid factor, SSA/Ro, SSB/La, and anti-ANA antibodies. These autoantibodies have the potential to target self-tissues, specifically the lacrimal and SGs, leading to inflammation and tissue damage (100–102). In the presence of genetic and epigenetic abnormalities, external stimuli such as viruses, infections, trauma, and environmental factors can trigger specific subsets of B cells, notably marginal zone (MZ) B cells, plasmablasts, and plasma cells. This process of activation results in the generation of autoantibodies and immune complexes, thereby initiating autoimmune reactions in tissues (103). Research has shown an elevation in B cell subsets in individuals with SS, characterized by heightened CD19 expression that potentially triggers B cell receptor signaling (104). Furthermore, a notable decrease in circulating CD27+ memory B cells is found in SS sufferers’ peripheral blood, contrasting with their increased presence in the SGs (105). There is also evidence indicating that B cells significantly assist in the pathogenesis of SS in individuals displaying ectopic germinal center-like structures within their exocrine glands (106) (**Figure 2**).

### 3.1 Plasmablasts and plasma cells

Plasmablasts, an early differentiated form of plasma cells also referred to as long-lived plasma cells, originate from B cells in the course of immune responses. Possessing a notable proliferative potential, plasmablasts ultimately develop into plasma cells, which generate substantial quantities of antibodies to counteract foreign antigens (107). Researchers employing cytometry time of flight (CyTOF) technology unveiled alterations in B cell subsets among individuals with SS. Plasmacytoid dendritic cells (pDCs), CD4+ T and CD27+ memory B cells were less prevalent, whereas the quantity of activated CD4+ and CD8+ T cells and plasmablasts demonstrated an increase. Furthermore, the escalation of plasmablasts in the bloodstream and plasma cells in the SGs of individuals with SS is positively connected to serum IgG levels, disease activity, and autoantibody positivity (108, 109). The decreased presence of CD27+ memory cells may be credited with their heightened differentiation into plasma cells. Aqrawi et al. observed heightened levels of CD27-expressing plasmablasts and plasma cells in SGs (110), while Hansen et al. noted a notable rise in CD27+ B cells in inflammatory tissues (111). Additionally, a study on systemic lupus erythematosus (SLE) patients identified a mucosal phenotype of circulating plasmablasts that express the chemokine ligand CCL28, release IgA and IgG, and move to mucosal locations (112). Given the mucosal affinity of SS and its resemblance to SLE, it is plausible that IgA and IgG autoantibody-producing plasma cells participate in the advancement of SS. Approximately fifty percent of the B cells present in the peri-lobular stroma of SG tissue specimens are mature plasma cells. Furthermore, investigations on SS patients exhibiting renal complications underscore the significance of plasma cell infiltration in SS-related interstitial nephritis (113, 114).

### 3.2 Marginal zone B cells

Marginal zone B cells (MZB) are a distinct subset of B lymphocytes predominantly situated within the splenic marginal region, where they serve a crucial function in promptly reacting to circulating pathogens and generating innate antibodies (115). Additionally, MZB cells can be identified in lymph nodes and blood, and are essential contributors to innate immune responses. Activation of MZB cells can occur through antigen recognition by the B cell receptor (BCR) facilitated by dendritic, neutrophil, and reticular cells, or through toll-like receptor (TLR) stimulation by macrophages (116). Recent research has presented that MZB cells accumulate in the SGs of individuals with SS and assist in glandular destruction through the production of autoantibodies (117). This phenomenon has also been observed in SS mouse models, where MZB cells demonstrated a notable rise in both the spleen and SGs (118). Experimental studies using transgenic SS mouse models have demonstrated that the elimination of MZB cells led to normal saliva secretion and preserved SG histology (119). Non-Hodgkinlymphoma, a serious complication in SS patients, is primarily derived from MZB cells, underscoring the significance of this subset in SS (120).

### 3.3 Regulatory B cells

Regulatory B cells (Bregs) are a particular subgroup of B lymphocytes known for their immunomodulatory properties, primarily characterized by the secretion of cytokines like IL-35, IL-10, and Granzyme B(GrB) to inhibit the activity of other immune cells, thereby taking part in the control of excessive inflammatory responses and influencing disease progression (121). Previous research indicates that different subsets of Bregs may exhibit shared surface markers. For example, Mauri et al. have identified Bregs with the phenotype CD19CD24^+hi^CD38^hi^, while other studies have observed an enrichment of IL-10-generating B cells within the CD24hiCD27 B cell population (122, 123). Initial research suggested that Bregs can suppress immune responses mediated by Th17 cells and improve collagen-induced arthritis through the manufacturing of IL-10. Furthermore, Bregs have been shown to hinder the proliferation of Th1 cells by promoting the expansion of Tregs (122, 124). Recent studies have identified an inverse relationship between IL-10-secreting Bregs and Tfh cell responses. As SS advances, the frequency of Breg cells tends to decrease while the quantity of Tfh tends to increase (125). Additional cytokines participate in the regulatory functions of B cells. IL-35, a recently discovered cytokine consisting of a heterodimer of P35 and EBI3, has been identified as having potential regulatory functions (126). Bregs release IL-35 to directly suppress Th1 and Th17 cells and promote the proliferation of Tregs (127). In patients with SS, A reduction in serum IL-35 levels and a rise in IL-12 levels indicate an imbalance between pro-inflammatory and anti-inflammatory states (128). Furthermore, higher levels of IL-35 are linked to reduced disease activity in SS, underscoring the significance of IL-35-producing Bregs as SS developed.

### 3.4 BAFF and secondary lymphoid GCs

Th1 (BAFF), also known as CD257 or tumor necrosis factor ligand superfamily member 13B (TNFSF13B), is predominantly synthesized by myeloid cells. It has a critical role in controlling the proliferation, maturation, and survival of B lymphocytes, and is recognized as a critical factor in both local and systemic autoimmunity (129). Elevated levels of BAFF have been identified in rheumatic disorders including SLE and RA, with the most pronounced increase observed in patients with SS. B-cell malfunction and the development of autoantibodies are the hallmarks of these illnesses (52). Research has shown that SS patients exhibit heightened amounts of BAFF in both serum and SGs, with a notable association between the levels of BAFF and the antibodies against SSA/Ro and SSB/La (130). While BAFF is typically generated by monocytes, macrophages, and dendritic cells, SS patients demonstrate BAFF secretion by T cells, B cells, and salivary epithelial cells (131).. Elevated BAFF levels contribute to an increase in B-cell subpopulations resembling MZB cells within the exocrine glands. Conversely, decreased BAFF levels can prompt the differentiation of IL-10-producing Bregs (132). Ectopic lymphoid structures resembling secondary lymphoid organ GCs have been identified in around 25-30% of labial and parotid glands of patients with SS. B cells that produce autoantibodies are situated at the periphery of these GC-like structures, potentially playing a role in persistent B-cell activation (133). The development of GCs is reliant on the presence of chemokines CXCL12 and CXCL13, which have been prominently detected in the SG tissues of individuals with SS (134). The importance of CXCL13 in the development of ectopic lymphoid tissues is underscored by its binding to the CXCR5 receptor on B cells, which, in conjunction with BAFF, facilitates the aggregation of CD27+ memory B cells in the SGs (135). Furthermore, the microenvironment of patients with SS promotes the formation of ectopic lymphoid tissues and germinal centers, with these GC-like structures exhibiting heightened focal infiltration and a correlation with disease severity.

## 4 Discussion

The pathogenesis of SS involves a multitude of immune cells, with T and B lymphocytes, along with their respective subgroups, playing pivotal roles in the disease’s mechanism. The intricate relationship between these cells collectively drives the progression of the condition (136). Antigen-driven, T cell-mediated hyperactivity of B cells, which results in autoreactive B cell activation and the development of ectopic germinal centers and B cell lymphomas within the SGs, is a defining feature of SS. In these glands, effector B cells enhance the growth as well as multiplication of CD4+ T cells through a beneficial feedback cycle. Activated B cells internalize antigens via the B cell receptor (BCR), subsequently processing peptides in a class II MHC-independent manner to present to CD4+ T cells, thereby modulating both the reactions of primary and memory CD4+ T cells (137). Among the T cell subgroups implicated in SS, Tfh stimulate B cell responses that are dependent on T cells inside GCs, primarily by secreting IL-21, which induces the activation of B cells and their development into plasma cells (138). Conversely, regulatory B cells that produce IL-10 inhibit Tfh cell responses in SS, indicating that fine-tuning of B cell responses is crucial for controlling autoimmunity and T cell reactions in the syndrome. Additionally, Tfr exert an immunosuppressive effect on the proliferation and activation of Tfh and B cells within secondary lymphoid tissues (139).

Since T cells have long been known to play a major role in SG damage in SS, focusing on T cell-associated cytokines offers a potentially effective treatment strategy. A special biologic called abatacept (CTLA4-Ig) attaches itself to CD80/CD86 on APCs, inhibiting the co-stimulatory molecules required for full T cell activation. Additionally, it lowers the quantity of cTfh and the degree to which T cells express inducible co-stimulatory molecules, which inhibits the activities of both CD4+ and CD8+ T cells (140). It has been demonstrated that the connection between the transmembrane glycoprotein CD40 on APCs and B cells and its ligand CD40L on activated CD4+ T cells enhance cellular and humoral immune responses. Elevated levels of CD40L in SS individuals and heightened transcripts of CD40L in CD4+ T cells highlight the potential of targeting CD40 with the inhibitory monoclonal antibody Iscalimab as a viable treatment option for SS (141). SG epithelial cells from SS individuals demonstrate a high degree of the inducible co-stimulatory molecule ligand (ICOSL), which, in conjunction with IL-6, promotes the precise distinction of activated CD4+ T cells into follicular T cell subgroups, subsequently secreting IL-21 to induce B cell activation. Prezalumab, a fully humanized antibody targeting ICOSL, has been used in a recent placebo-controlled study to treat patients with active primary SS (142). RO5459072 is a covalent, reversible, and selective inhibitor of protease S, expected to reduce antigen presentation mediated by MHC-II and attenuate the activation of CD4+T cells. This is likely to inhibit the production of T-cell-dependent autoantibodies and neutralize tissue damage caused by activated macrophages and neutrophils (143). A randomized, double-blind, parallel-group clinical study found that treatment with RO5459072 slightly reduced B and CD8+T cells in SS patients, yet there was no significant improvement in disease activity and symptoms (144).

In experimental SS mouse model, intra-glandular injection of anti-CD103 monoclonal antibody effectively reduces tissue-resident memory T cells, alleviates gland damage, and enhances salivary function. These findings highlight the potential of T cell-related cytokines as therapeutic targets for SS, offering a strategy to control disease progression through T cell modulation (145). Additional, anti-CD4 monoclonal antibody eye drops reduce activation and proliferation of specific CD4+ T cells in the mouse SS model and prevent disease progression in the lacrimal glands (146). Intranasal administration of the autoantigen α-fodrin reduces serum IFN-γ levels and exocrine gland infiltration, expands peripheral FoxP3+ Tregs, delays autoantibody production, and prevents the exacerbation of exocrine gland disease in NOD mice (147). KPL-404 is a humanized monoclonal antibody targeting the crucial CD40-CD40L pathway for B cell activation, serving as an effective therapeutic for SS. By blocking CD40 on B cells, KPL-404 disrupts the interaction between B cells and CD40L on T cells, thereby inhibiting the activation and proliferation that lead to SS lesions. KPL-404 effectively reduces pathogenic B cell responses without causing B cell depletion or triggering adverse immune activation (148). Preclinical studies indicate that KPL-404 blocks downstream NF-κB activation mediated by CD40, inhibits primary and secondary antibody responses, and exhibits favorable pharmacokinetic properties. These research findings have propelled the clinical development of KPL-404 as a targeted therapy for SS (149).

Similarly, therapies targeting B cells are attracting increasing attention. The transmembrane protein CD20, which is found on mature and pre-B cells but not on progenitor B cells or normal plasma cells, has been the subject of the most research in B cell biology. Currently, the anti-CD20 monoclonal antibody rituximab is being investigated in SS, with studies indicating its efficacy in alleviating dryness symptoms and glandular manifestations in patients, and rapidly depleting B cell subpopulations in peripheral blood as well as SGs (150). In addition, rituximab downregulates T-cell subsets associated with GC formation and B-cell activation, suggesting a potential therapeutic effect of rituximab in SS (151). CD22 is a B cell-restricted transmembrane sialoglycoprotein, appearing late in pre-B cells, little expressed in developing B cells, and intensely articulated in mature B cells but nonexistent in differentiated plasma cells. Epratuzumab, a monoclonal antibody against CD22, has shown effectiveness in reducing B cell numbers in patients with SLE without increasing adverse events, suggesting its potential as a novel therapeutic for SS, pending validation through extensive clinical trials (152). Treatment with anti-Ly9 (CD229) monoclonal antibodies in an SS mouse model selectively depletes pathogenic B cell subsets, including B1, MZB, and GC B cells. This targeted depletion reduces lymphocytic infiltration in the salivary glands and kidneys, lowers autoantibody levels, and effectively mitigates the glandular and extra-glandular manifestations of SS (153). Blocking the IL-7 receptor α-chain with an antibody in newly diagnosed female NOD mice with SS significantly improves the disease pathology, which correlates with reduced production of IFN-γ by CD4+ T cells, CD8+ T cells, and B cells in the SMG, along with decreased levels of chemotactic lymphocyte attractants CXCL9, -10, -11, and -13 (154).

As previously mentioned, the BAFF is essential for B cell activation, survival, and differentiation, and targeting BAFF regulation may offer new hope for treating SS patients. Belimumab, a monoclonal antibody targeting BAFF, significantly reduces ESSDAI, ESSPRI, and average dryness VAS scores in SS patients, with most B-cell biomarkers (including IgG, IgA, IgM, free κ, and λ light chains), RF titers, and average B-cell counts showing improvement (155). Another study indicates that Belimumab treatment significantly reduces immature B-cell subpopulations in SS and normalizes BAFF-R (BAFF receptor) expression across all B-cell subpopulations (156). Discontinuation of Belimumab is reported to facilitate relapse in SS (157). Lanalumab (VAY736), a human IgG1/κ monoclonal antibody targeting human BAFF-R, has been studied for its potential in treating SS. A current single-center, double-blind, placebo-controlled phase II study of Ianalumab shows positive therapeutic effects on SS, with Ianalumab also capable of depleting B cells through antibody-dependent cellular cytotoxicity (ADCC) (158). Iguratimod, a novel anti-rheumatic drug, targets the key regulatory kinase TEC in B cell function, inhibiting BAFF-induced B cell activation and plasma cell differentiation, thereby reducing autoantibody production and more effectively alleviating SS symptoms (159). This study reveals the potential of iguratimod in treating SS by inhibiting TEC function, offering a treatment strategy distinct from traditional kinase inhibitors.

The pathogenesis of SS involves the complex dysregulation of multiple immune pathways. Compared to single-target therapies, targeting these pathways in combination may offer a more effective treatment approach. *In vitro* studies using peripheral blood mononuclear cells from healthy controls and SS patients have shown that the combined use of leflunomide (LEF) and hydroxychloroquine (HCQ) can dose-dependently inhibit the proliferation of T and B cells, and reduce levels of key pro-inflammatory cytokines (CXCL13, IFN-α, IFN-γ) and immunoglobulins (IgG, IgM) associated with SS pathology. Notably, HCQ exhibits a potent B-cell-specific inhibitory effect, while LEF is more effective at inhibiting T-cell activation (160). The observed synergistic effects at clinically achievable concentrations suggest that this dual therapy could provide a novel and comprehensive strategy for managing SS by addressing its complex immunological underpinnings. In conclusion, further research into the identification and functional characterization of novel T and B cell subgroups within the SS pathogenesis will aid in developing new therapeutic strategies for SS and other autoimmune diseases. Additional translational research is needed to validate the feasibility and efficacy of these methods in clinical settings.
